# Psychological skills training and a mindfulness-based intervention to enhance functional athletic performance: design of a randomized controlled trial using ambulatory assessment

**DOI:** 10.1186/s40359-016-0147-y

**Published:** 2016-07-26

**Authors:** Philipp Röthlin, Daniel Birrer, Stephan Horvath, Martin grosse Holtforth

**Affiliations:** 1 Swiss Federal Institute of Sport, Alpenstrasse 18, CH-2532 Magglingen, Switzerland; 2 University of Zürich, Zürich, Switzerland; 3 University of Bern, Bern, Switzerland; 4 Psychosomatic Competence Center, Inselspital Bern, Switzerland

**Keywords:** Sport psychology, Intervention, Randomized controlled trial, Psychological skills training, Mindfulness, Performance enhancement, Elite sport, Athletic performance, Ambulatory assessment

## Abstract

**Background:**

Struggling to deliver performance in competitions is one of the main reasons why athletes seek the advice of sport psychologists. Psychologists apply a variety of intervention techniques, many of which are not evidence-based. Evidence-based techniques promote quality management and could help athletes, for example, to increase and maintain functional athletic behavior in competitions/games (i.e., being focused on task relevant cues and executing movements and actions in high quality). However, well-designed trials investigating the effectiveness of sport psychological interventions for performance enhancement are scarce.

The planed study is founded by the Swiss National Science Foundation and examines the effectiveness of two interventions with elite and sub-elite athletes. A psychological skills training (PST) and a mindfulness-based intervention (MI), administered as group-program, will be compared to a waiting-list control group concerning how they enhance functional athletic behavior - which is a prerequisite for optimal performance. Furthermore, we will investigate underlying mechanisms (mediators) and moderators (e.g., task difficulty, individual characteristics, intervention-expectancy and intervention-integrity).

**Methods/Design:**

The presented trial uses a randomized controlled design with three groups, comparing PST, MI and a waiting list control condition. Both group interventions will last 5 weeks, consist of four 2 h sessions and will be administered by a trained sport psychologist. Primary outcome is functional athletic behavior assessed using ambulatory assessment in a competition/game. As secondary outcomes competition anxiety, cognitive interference and negative outcome expectations will be assessed. Assessments are held at pre- and post-intervention as well as at 2 months follow up. The study has been approved by the ethical committee of the Swiss Federal Institute of Sport.

**Discussion:**

Both PST and MI are expected to help improve functional behavior in athletes. By examining potential mechanisms of change and moderators of outcome we will not only be able to answer the question whether the interventions work, but also how, under what conditions, and for whom. This study may also fill a gap in sport psychology research, considering the current lack of randomized controlled trials. In the future, researchers could use the presented study protocol as template to investigate similar topics in sport psychology.

**Trial registration:**

ISRCTN11147748, date of registration: 11 July 2016.

## Background

Sport psychologists try to teach athletes how to perform optimally on the highest possible level even under challenging and non-optimal conditions. Performing optimally means that athletes are able to deliver 100 % of their performance at one specific point in time. We consider functional behavior in this context as necessary, but not sufficient, for optimal performance. *Functional athletic behavior* (FAB) is characterized by a high quality of actions and movements and an attentional focus on relevant performance cues or valued distal goals (e.g., “the ball” or “being a fair sportsmen”, adapted from Gardner and Moore [23, 24]). Factors that may negatively influence FAB include, for example, negative outcome expectations, too much or not enough autonomic arousal, or an attentional focus on external and internal threats (e.g., strong negative emotions like anxiety) or on irrelevant cues (e.g., task irrelevant thoughts and worries [23, 24, 64]).

To help athletes increase and maintain FAB, sport psychologists may use a wide range of interventions, which can be grouped into two main categories. Traditionally, (1) *psychological skills training* (PST) has been the most common intervention of choice [74]. Recently, (2) *mindfulness-based interventions* (MI) have been proposed as an alternative in sports [7, 26]. In this study we aim to compare the effect of PST and MI on FAB and to examine the underlying mechanisms of these interventions. In addition, we aim to show the suitability of FAB as a construct to evaluate sport psychological interventions and the utility of our FAB measure as an alternative outcome variable, which solves some of the problems of frequently used objective measures of performance.

## Psychological skills training

PST encompasses a set of techniques, namely *self-talk*, *imagery*, *goal setting*, and *arousal regulation* [33, 74]. *Self-talk* is the “syntactically recognizable articulation of an internal position that can be expressed either internally or out loud, where the sender of the message is also the intended receiver” ([73], p. 140). Self-talk can be instructional (e.g., “look at the ball”) or motivational (e.g., “I will run to the finish with all the energy I have”). Intentionally regulating self-talk may increase FAB because it could help athletes to remind themselves of key skills and strategies and to direct their attention and behavior accordingly [32, 73].

*Imagery* describes the process by which existing information from memory (e.g., of a movement) or newly generated images are vividly and deliberately experienced or re-experienced. This process involves *all* one’s senses and may occur in the absence of a real stimulus (e.g., a skier imagines racing a downhill course [53]). Imagery may help athletes to behave functionally because it could facilitate the recollection of corresponding psychological states and movements [27].

Scholars differentiate three types of goals that can be specified and monitored in *goal-setting*: *outcome goals* are defined as the final result or outcome of a competition or a game (e.g., a rank or winning and losing); *performance goals* are evaluated on the basis of a comparison between an athlete’s own previous achievements rather than an opponent’s performance (e.g., improving one’s passing accuracy from 70 to 80 % or increasing one’s first serve percentage compared to the last tournament); and *process goals* focus on how an athlete performs a certain skill by clarifying what actions have to be in mind in order to execute that skill at an optimal level (e.g., a gymnast focussing on having the correct posture and amount of tension in the body [77]). The latter two may increase FAB by helping athletes to know what they need to do and to direct their attention and behavior accordingly. Furthermore, making athletes aware of their own standards compared to their ongoing performance might motivate athletes to increase their effort and persistence [48].

*Arousal regulation* includes all techniques that influence physiological arousal by either decreasing (e.g., breathing or bodily relaxation techniques) or increasing it (e.g., breathing techniques or behaving in physically arousing ways [1]). The optimal extent of physiological arousal depends on the type of sport (e.g., it is lower in pistol shooting than in weight lifting), task difficulty, individual preference [44], and current psychological states (e.g., cognitive state anxiety [31]). Athletes who are able to adapt their arousal to perceived deviations from an ideal degree might be less distracted and thus have a greater chance of behaving functionally.

## Mindfulness-based interventions

MI refer to interventions that foster mindfulness. *Mindfulness* describes the ability to hold one’s attention on momentarily experienced bodily sensations, acoustic and visual perceptions, emotions, or thoughts and to observe them in an accepting and compassionate manner without automatically reacting to or elaborating on them [46]. MI have been found to enhance subcomponents of attention, such as orienting, conflict monitoring (especially in the early stages of mindfulness training), and alerting (in later stages [12, 70]).

Improvements in orienting (also referred to as selective attention or concentration, i.e., to limit attention to a selection of several sensorial stimuli), conflict monitoring (or divided attention or executive attention, i.e., to prioritize among competing thoughts, feelings and behavioral responses), and alerting (or sustained attention or vigilance, i.e., to attain and hold an alert state of readiness [12, 57]) may increase and maintain FAB by helping athletes to concentrate on the task at hand, in the presence of potential internal and external distractors, and over a long period of time.

Besides the desirable effects of MI on attention, research showed that they led to an increase in acceptance of unpleasant experiences (e.g., negative thoughts and emotions or bodily sensations, [47]). Accepting means that rather than avoiding negative experiences, one exposes oneself to such experiences without trying to change or control them. This may make FAB more likely because trying to change emotions and thoughts in maladaptive ways (e.g., ruminating, worrying, or experiential avoidance [37]) could bind attentional resources needed for the current athletic task at hand [23]. Also, intending to consciously suppress negative thoughts often contains the object to be avoided and thus ironically has a greater chance of influencing behavior (e.g., trying not to think about hitting the golf ball in the bunker involves the image of hitting the ball in the bunker, making the correspondent behavior more likely [42, 76]).

In addition to improved attention and an increased acceptance, defusion might be another mechanism by which MI increases and maintains FAB. Defusion has been found to be increased after MI [18, 34] and describes the ability to observe one’s thoughts and emotions and view them as passing mental events rather than identifying with them [38]. This is in line with findings showing that mindfulness promotes the ability to quickly let go of negative thoughts [21]. Defusion might help athletes to behave functionally because they would no longer act automatically and would be flexible in dealing with (negative) thoughts and emotions. In this context “flexible” means being able to decide when and when not to follow an (emotional) impulse. An athlete’s behavior would therefore not be determined by certain potentially performance-inhibiting states like anxiety [62].

While there are reasons to assume that both PST and MI promote FAB, they probably do so in different ways. PST is based on the assumption that the development of self-control of internal states, such as thoughts, emotions, and physical experiences, enhances athletic performance. In contrast, MI assumes that athletes’ performance benefits by altering how they relate to their experiences (i.e., to control vs. to accept and defuse). These differing assumptions represent the different theoretical origins of PST and MI within cognitive-behavioral psychology, that is classical vs. “third-wave” approaches [6, 22, 35].

## Effectiveness of PST and MI randomized controlled trials on athletic performance

There is evidence from case studies and correlational research that use of self-talk, imagery, goal-setting, arousal-regulation, and mindfulness are all related to objectively measured athletic performance or performance-related psychological variables (see [24, 63], for an overview). However, the effects of PST and MI on FAB have not been investigated so far, and there are hardly any randomized controlled trials (RCT) investigating PST packages (i.e., a set of multiple PST methods) or MI in adult athletes [24, 63]. Given their potential to test causal hypotheses, RCTs can be considered the gold standard in intervention research.

To our knowledge, there are five RCTs investigating the effects of a combination of several PST (i.e., at least two or more techniques) on performance or performance-related psychological variables in adult athletes (i.e., at least 18 years old), two of which found no differences in outcome (objective measures of performance, i.e., *pass efficiency*, *on target accuracy in competition*, *service percent*) between intervention- and waiting-list control group [49, 56]. The other three found improvements in outcome (objective and subjective measures of performance, i.e., *runs scored*, *wickets taken*, *neuro-muscular performance*, *blinded coach ratings of performance*, and *coach-rated performance consistency*; and performance-related psychological variables, i.e., *use of psychological skills* and *anxiety*) in the intervention groups compared to contact-control groups [14, 40, 71]. The PST interventions showed medium to large effect sizes, a range between 0.5 and 12 contact hours and lasted between 10 days and 4 months.

To our knowledge, there are four RCTs investigating the effects of MI on performance or performance-related psychological variables in adult athletes, all of which found improvements in outcome (objective measures of performance, i.e., *shooting performance*, and performance-related psychological variables, i.e., *mindfulness*, *flow*, *stress*, *competition anxiety*, and *pessimism*) in the intervention group compared to waiting-list [2, 67] or not specified control groups [43, 52]. The MI interventions showed medium to large effect sizes, a range between 0 (disposal of an information sheet) and 8 contact hours and lasted between 4 and 8 weeks.

In sum, current research suggests that both, PST and MI may somewhat promote performance or performance-related psychological variables, especially when the intervention lasts for several weeks and involves some form of daily practice. However, several critical points related to these studies need to be kept in mind. There are only a few studies, some of which deal with power issues (i.e., number of subjects too small to detect effects) and quality issues (e.g., no active control group; no manipulation check, i.e., whether PST and MI lead to greater use of psychological skills and mindfulness, respectively; time spent practicing psychological skills or mindfulness not recorded; treatment adherence not evaluated). Only one study [71] investigated the impact of all four psychological skills described above (however, this study is quite promising). Furthermore, is it difficult to compare the above studies because they examine the effect of different forms of PST and MI of varying duration on various outcome variables (i.e., objective or subjective measures of performance, such as scores or coach ratings, respectively, or performance-related psychological variables, such as flow or anxiety). Given its central importance for the evaluation of sport-psychological interventions, the general use of outcome variables needs to be examined in more detail before proceeding to our study protocol.

## FAB as an alternative outcome variable in sport psychological interventions?

Because improving performance is in the center of coaches’ and athletes’ interest, objective measures of performance are often used as the major outcomes when scientifically examining the effect of sport psychological interventions. Notwithstanding the importance of the ultimate success, athletic performance is influenced by a wide range of potentially interfering factors, such as actual training load, being in shape, injuries, weather, the opponent, whether an athlete is in a preparation or competition phase, etc. Thus we argue that objective measures of performance are too distant to determine whether a certain sport psychological intervention was successful. In that regard, measuring whether an intervention promotes FAB is preferable because it is less dependent on interfering factors (i.e., it is possible to perform functionally, for example, when not in shape), allows for a comparison between different sports, and is very close to what athletes actually do in games or competitions (as opposed to, for example, what has to be done in an experiment). In the method section, we will present how we intend to operationalize FAB.

## Current study

This study tries to bridge some of the gaps in the current research by conducting a high-quality RCT based on the CONSORT criteria [66], comparing a PST, a MI, and a waiting-list control group. We will use an outcome variable (FAB) that allows us to compare different sports and allows for examining the success of the interventions regardless of external factors, such as being in shape or training load. This way, we will be able to determine whether PST and MI are effective. Furthermore, by investigating the role of mediators and moderators, we will gather evidence about mechanisms of change and for whom each intervention is most beneficial. Our primary interest is to determine the effect of PST and MI on FAB. In order to examine the effects of a broader range of outcomes, factors that may negatively influence FAB (i.e., negative outcome expectations, competition anxiety, and cognitive interference) and objective and subjective measures of performance will be assessed as secondary outcome variables.

### Mediators

To investigate *how* PST and MI may promote functional athletic behavior we will examine underlying mechanisms of change and to what degree these mechanisms are specific or shared by PST and MI. In order to do this, we examine mediators of PST and MI and analyze differential predictors of the two. Grounded on the theories underlying PST and MI, the following three groups of mediators were selected: (1) *Mediators specific to PST* (i.e., use of psychological skills as a manipulation check of the PST intervention and the ability to control thoughts and emotions); (2) *mediators specific to MI* (i.e., mindfulness as a manipulation check for the MI intervention and the ability to accept and defuse from thoughts and emotions); and (3) *mediators assumed to play a role in both interventions* (i.e., general attention, attention control in games, and competitions).

### Moderators

To our knowledge no studies have examined factors potentially moderating the effectiveness of PST or MI. Such information is of crucial importance as it may specify the influence of certain situational variables or identify groups of individuals likely to either benefit from an intervention or not. In order to examine for whom and under which conditions PST and MI are (not) effective, three types of moderators are investigated in the present study. Most importantly, we will look at *situational variables*, i.e., task difficulty and the importance of the game or competition for the individual athlete. The other two kinds of moderators can be divided into *basic demographic factors* (e.g., age, gender, kind of sport, performance level, and previous experience with PST and MI), and *individual characteristics* that research has identified as being relevant in situations of athletic performance like task- and ego-orientation [16], self-esteem [50], or self-compassion [3, 19, 54].

### Study objectives and hypotheses

The primary objective of this study is to assess the effects of PST and MI in promoting FAB in elite athletes. We hypothesize that both active interventions are more effective than a waiting-list control condition in promoting FAB. We do not expect PST to be superior over MI or vice versa; rather, they are expected to increase FAB through different routes.

Thus, the secondary objective is to examine potential factors that mediate the effects of PST and MI. We assume that the effect of PST on FAB is mediated by the use of psychological skills (the manipulation check of the PST intervention) and the experienced ability to control one’s thoughts and emotions. We expect the effect of MI on FAB to be mediated by participants’ self-rated mindfulness (the manipulation check of the MI intervention) and acceptance of/defusion from unpleasant experiences. Finally, we hypothesize the effects of both PST and MI on FAB to be mediated by general level of attention and perceived attention control in games or competitions.

A third objective is to examine potential moderators of the effects of PST and MI. We assume that the more athletes interpret their current experience as something that has to be regulated, the more they profit from regulation or coping strategies being utilized/taught in PST and MI. We therefore hypothesize that athletes high in ego orientation or those with low values of self-esteem and self-compassion benefit the most from PST and MI. Such athletes might perceive performance situations as threatening for their psychological needs to a greater chance [24, 28], especially when the athletic task at hand is difficult or the competition is perceived as important. Perceived threat might lead to, for example, more competition anxiety, negative outcome expectations, or cognitive interference. In addition, we aim to investigate the role of treatment expectancies (common factor) and treatment adherence for improvements in FAB.

## Method

### Participants and power analysis

The target group are elite, sub-elite, and recreational athletes from four sports (curling, volleyball, i.e., indoor and beach, tennis, and hockey, i.e., floorball and ice hockey). We chose these sports because they are suited to sample comparable short sequences (see primary outcome below) within a game or a competition, as opposed to, for example, soccer. Athletes who are members of the respective Swiss national sport associations will be contacted and offered the opportunity to participate. Criteria for exclusion are a likely mental disorder, significant previous experience with PST or MI, less than 4 h of athletic training per week, or being younger than 18.

The sample size calculation is based on differences between the waiting-list control group (WC) and one of the treatment groups (PST or MI) after the intervention. Based on previous research, we assume medium effect sizes of d = .6 [39]. Testing one-sided, given that α = .05 and a power of 80 %, we would need 108 participants (36 for each group) to be able to detect the effect.

### Study design and group allocation

**Fig. 1:**
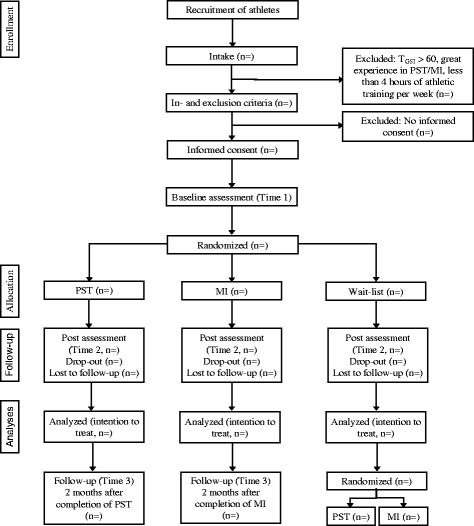
Participant recruitment and flow through the study

Figure 1 shows an overview of the procedure (parallel group design). After a first contact and checking for inclusion and exclusion criteria (time 0), athletes will be stratified for gender, sport, and performance level, then randomly assigned to either the PST group, the MI group, or the WC group, and will be informed about their experimental condition. Members of the same team (i.e., curling, volleyball, or hockey) will be assigned to the same intervention group. For randomization, a computer-generated random-number sequence will be prepared in advance and sealed in opaque, consecutively numbered envelopes by an independent researcher. An independent researcher will open the envelopes in sequence based on client number, to determine the participant’s assignment to the groups.

Table 1 gives an overview of the assessment/measures. PST and MI participants will be assessed at pre-intervention (time 1), post-intervention (time 2), and at 2 months follow-up (time 3). WC participants will be assessed at time 1 and 2, and will then be randomly assigned to the PST or MI intervention. Participants will complete a series of questionnaires at all assessment time points. Participants names will be coded in the data file for anonymization and the code key will be stored in a different file. Since it is not possible to mask condition assignment for the participants or the experimenter, we will assess and control for outcome expectations regarding the assigned intervention in order to control for potential effects on outcomes (see recommendations by Boot et al. [10]).

**Table 1:** Instruments assessing inclusion and exclusion criteria, primary/secondary outcomes, moderators, mediators, and common factor

Concept	Measurement (items)^a^	Time points^b^			
		T0	T1	T2	T3
Inclusion/exclusion criteria					
Clinical level of psychopathology	BSI-18 (18)	x			
Experience with PST/MI	-	x			
Primary outcome measure					
Functional athletic behavior	Ambulatory assessment		x	x	x
Secondary outcome measures					
Psychological variables					
State anxiety	CAI-S (scales cognitive and somatic anxiety, 8)		x	x	x
Cognitive interference	TOQS (17)		x	x	x
Negative outcome expectation	CAI-S (confidence scale inversed, 4)		x	x	x
Athletic performance					
Objective measures	E.g., win/lose, points scored		x	x	x
Subjective measures	Self-rated measures of performance		x	x	x
Mediators					
Use of psychological skills	TOPS (scales self-talk, imagery, goal-setting, relaxation and activation, 20)		x	x	x
Ability to control thoughts and emotions	TOPS (scales negative cognitions and emotional control, 8)		x	x	x
Mindfulness	FFMQ-SF (24) & AMQ (16)		x	x	x
Acceptance of (unpleasant) experiences	SEC-27 (acceptance scale, 3) & AAQ-II (inversed, 9)		x	x	x
Defusion	EQ (decentering scale, 7), DSS (12)		x	x	x
General attention	ANT		x	x	x
Moderators					
Task difficulty	-		x	x	x
Importance of game/competition	-		x	x	x
Demographic characteristics	-	x			
Task- & ego-orientation	TEOSQ (13)		x		
Self-esteem	RSC (10)		x		
Self-compassion	SCS short form (12)		x		
Common factor & practice time					
Athletes’ expectancy of the intervention	- (3)		x		
Practice time	Practice sheets			x	x

^a^ *BSI* brief symptom inventory, *CAI-S* competition anxiety inventory state, *TOQS* thought occurrence questionnaire sport, *TOPS* test of performance strategies, *FFMQ-SF* five facets mindfulness questionnaire short form, *AMQ* athletic mindfulness questionnaire, *SEC-27* self-assessment of emotional competencies, *AAQ-II* acceptance and action questionnaire, *EQ* experience questionnaire, *DSS* decentering scale for sport, *ANT* attention network test, *TEOSQ* task ego orientation sport questionnaire, *RSC* rosenbergs’s self-esteem scale, *SCS* self compassion scale

^b^T0 = Before randomization T1 = pre-intervention, T2 = post intervention, T3 = 2 months follow up

### Description of the PST and MI intervention

The PST intervention will be adapted from PST programs like Ebersbächer’s *Mental Training* [15]. It involves the practice of four psychological skills (self-talk, imagery, goal-setting, and arousal control) and will be based on the latest guidelines and recommendations about instruction and application of these skills [1, 27, 73, 77]. A sport psychologist trained in specialized PST interventions will explain the expected sport-specific benefits to the participating athletes and advise athletes to do homework between group sessions.

The MI will be adapted from mindfulness-intervention programs, including Mindfulness-Acceptance-Commitment (MAC) [25], Acceptance and Commitment Therapy (ACT) [38], and Mindfulness-Based Stress Reduction (MBSR) [45]. A sport psychologist trained in specialized mindfulness-based interventions will explain the expected sport-specific benefits to the participating athletes and advise athletes to do formal and informal mindfulness practices at home between group sessions.

The interventions will be realized in groups of six athletes each, leading to six sub-groups in each condition (6 × 6 = 36 athletes, see power analysis). A manual will accompany the instructions for the interventions. All components of both interventions must be administered according to the manual’s specifications. Two independent raters will evaluate the adherence to each intervention on the basis of video recordings. In addition to psycho-education and practice at home guided by audio files that can be played on computers or portable devices such as smart phones. All participants will also be provided with a calendar of their exercises to tick off as they are completed, and daily text messages will remind them to practice their PST or mindfulness exercises. Each intervention consists of four 2-h sessions over the course of 5 weeks. Participants will be advised to practice daily and record their amount of practice. All sessions will conducted at the center for elite sports of the Swiss Federal Institute of Sport.

### Measures

#### Primary outcome measure

We will assess FAB as the primary outcome measure using an *ambulatory assessment*^1^ procedure, that samples subjective psychological variables in real time and the natural environment [72]. We will analyze three to four in-game/match sequences (S1 to S3 or S4, respectively) specific for each sport. In curling we will evaluate the first end (S1), the end before half-time (S2), the first end after half-time (S3), and the last end of the game (S4).^2^ In volleyball we will analyze the second and third set of one game^3^ by evaluating the first 5 points in each set (S1 and S3) and the last 5 points in each set (S2 and S4). In tennis we will analyze the first two sets of one match: the second and the third game^4^ (S1 and S3) and the last two games of the set or the tiebreak (S2 and S4). In hockey we will analyze each period (S1-S3) of one game.^5^

After each sequence we will evaluate whether athletes behaved functionally in the last sequence. We will ask the athletes themselves to rate the last end (curling), the last 5 points (volleyball), the last two games or the tiebreak (tennis), or the last period (hockey). It is common practice in other studies using ambulatory assessment to use only a few items to keep the interference of the measurement to a minimum (e.g., [75]). To assess FAB, athletes will rate the following questions from 0 (*no agreement*) to 100 (*total agreement*) on a tablet computer using a scroll bar:

1. Rate regardless of the result or outcome: In the *last sequence*, my movements and actions were of a high quality (precise, energetic, well timed, etc.).
2. Rate regardless of the result or outcome: In the *last sequence*, I was focused on the task.
3. Rate regardless of the result or outcome: In the *last sequence*, I behaved on the pitch/field/ice as the athlete that I would like to be.

Before the first assessment, athletes will be given instructions in training when and how to answer the questions and then answer the questions in an actual game/competition for familiarization.

#### Secondary outcome measures

Secondary outcomes are *negative outcome expectancies* (assessed directly before the game or match) as well as *somatic* and *cognitive competition anxiety* (directly after the game or match), which are all measured by the respective scales of the Competition Anxiety Inventory State (CAI-S) [11]. *Cognitive interference* (directly after the game or match) is measured by the Thought Occurrence Questionnaire for Sport (TOQS) [61]. In addition, we will assess objective (win/lose, performance indicators relevant for the sports discipline, such as shot percentages, points scored, winners/unforced errors, and goals/assists) and subjective (self-rated) measures of performance.

#### Mediators

We will use all these measurements in all three study groups to examine whether changes are specific for the expected groups. *Use of psychological skills* (manipulation check for PST) will be assessed by the subscales *self-talk*, *imagery*, *goal-setting*, *activation* and *relaxation* of the Test of Performance Strategies (TOPS) [65]. Being able to control emotions and thoughts will be measured by the TOPS subscales *emotional control* and *negative cognitions*.

We will use short form of the Five Facet Mindfulness Questionnaire (FFMQ-SF) [8] and the Athletic Mindfulness Questionnaire (AMQ), [80] to assess *mindfulness* (manipulation check for MI); the respective subscale of the Self-Assessment of Emotional Competencies (SEC-27) [4] and the Acceptance and Action Questionnaire II (AAQ-II) [9] to assess *acceptance*; and the *decentering* subscale of the Experience Questionnaire (EQ) [20] and the Decentering Scale for Sport (DSS) [79] to assess *defusion*.

The Attention Network Test (ANT) [17] will be used to assess *general attention* (i.e., *orienting*, *conflict monitoring*, and *alerting*). To assess perceived *attention control in games or competitions*, we will use the respective subscale of the TOPS.

#### Moderators

We will assess *task difficulty* by having participants rate their opponents’ performance after each sequence from 0 (*very bad*) to 100 (*very good*), by assessing the opponents’ ranking/seeding (lower, equal, higher), and the ongoing score. To assess the *importance of the game/match*, we will have the athletes rate the importance on a scale from 1 (*not important*) to 7 (*very important*) before the game or the match. In addition to basic demographic factors (i.e., age, gender, type of sport, performance level, and experience with PST and MI), we will assess *task and ego orientation* using the Task and Ego Orientation in Sport Questionnaire (TEOSQ) [59]; *self-esteem* using Rosenberg’s Self-esteem Scale (RSC) [13]; and *self-compassion* using the short form of the Self-Compassion Scale (SCS) [41, 58].

#### Inclusion criteria and common factor

We will use the short version of the Brief Symptom Inventory (BSI-18) [68] to assess clinical level of psychopathology. A total scale score of the BSI (*Global Symptom Index*, GSI) of T > 60 indicates a significant level of psychopathology. In psychotherapy, treatment expectancies have been shown to predict change in outcome variables (e.g., depression, [29, 55]). For that reason we will examine the associations between athletes’ expectancies (after the randomization) and subsequent changes in the primary and secondary outcome variables.

### Statistical analysis

All analyses will be conducted as intent-to-treat. To assess if randomization is successful in balancing demographic characteristics across the treatment groups, we will compare age, use of psychological skills, mindfulness, and performance level using Student’s t-tests. Two-way (group x time) repeated measures ANOVAs will be used to answer the question regarding whether the intervention (independent variable) had an effect on the primary and secondary outcome measures (dependent variables). Significant overall effects will be followed up with post hoc tests and contrasts between intervention programs (e.g., PST and MI together compared to the WC group). Significance levels will be set at *p* = .05. If there are significant differences between different intervention sub-groups, we will perform multilevel analyses. To test the mediation and moderation models, we will follow the requirements for mediation and moderation suggested by Hayes [36]. The first three authors of this manuscript will have access to the full dataset.

## Discussion

This study is the first to examine the effectiveness both PST and MI in the same randomized controlled trial. We will assess FAB as the primary outcome measure of this study; however, we also investigate psychological variables that may negatively influence FAB (i.e., competition anxiety, negative outcome expectations, and cognitive interference) and objective and subjective measures of performance as secondary outcome variables.

Both interventions are hypothesized to improve FAB and reduce the extent of psychological variables that may prevent FAB compared to a waiting-list control group. If the interventions are effective, both PST and MI may be considered empirically validated methods to help athletes behave functionally, which can be considered a prerequisite for performing optimally. This study may also fill a gap in sport psychology research, considering the current lack of randomized controlled trials. Besides investigating the effectiveness of the two interventions, the current study intends to examine potential mechanisms of change and moderators of outcome. Therefore, we will hopefully not only be able to answer the question whether the interventions work, but also how, under what conditions, and for whom.

While the question of how to perform optimally in competition or a game is not the only reason why athletes seek the advice of a sport psychologist,^6^ it is a very common, perhaps even the most common reason [30]. The frequency of this issue being presented underlines the importance of defining FAB as construct and operationalizing it as an outcome measure of sport psychological interventions. Assessing FAB might also help to solve the problem that in researching sport psychological interventions, an abundance of outcome variables is used, which makes it difficult to compare different sports. Also because objective parameters of athletic performance are heavily influenced by physical and external factors, using FAB as an outcome is an attractive alternative. The use of the ambulatory-assessment method has the advantage of being very close to what athletes are actually doing and experiencing in games or competitions (i.e., a high external validity). The critical points of this method include the fact that it is time-consuming and hardly explored in sport psychology. As in other forms of real live measurements [51], the chosen method should be sufficiently brief to minimize interference with athletes’ behavior and prevent jeopardizing optimal performance. In subsequent studies, one could expand the assessment of FAB via ambulatory assessment by having athletes rate video recordings of behavioral sequences within the ongoing competition. Real live measurement is an increasingly used assessment method that offers many additional research options in (elite) sport.

Future studies might use the presented study protocol to examine the effect of PST and MI on other psychological phenomena that may disturb or facilitate FAB and that are not part of the current study, for example, rumination over mistakes. As we assume FAB to be important in every sport (i.e., FAB is not sport-specific), investigating FAB in other sports (e.g., gymnastics, athletics, freestyle ski and snowboard, golf, or basketball) could be a next step. Furthermore, future research may gainfully examine other personality traits potentially influencing (i.e., moderating) athletic performance, such as perfectionism [69], narcissism [60], or intrinsic motivation [78].

## Abbreviations

AAQ-II, acceptance and action questionnaire; ACT, acceptance and commitment therapy; AMQ, athletic mindfulness questionnaire; ANT, attention network test; BSI, brief symptom inventory; CAI-S, competition anxiety inventory state; DSS, decentering scale for sport; EQ, experience questionnaire; FAB, functional athletic performance; FFMQ-SF, five facets mindfulness questionnaire short form; GSI, global symptom index; MAC, mindfulness-acceptance-commitment; MBSR, mindfulness-based stress reduction; MI, mindfulness-based interventions; PST, psychological skills training; RCT, randomized controlled trial; RSC, Rosenbergs’s self-esteem Scale; SCS, self compassion scale; SEC-27, self-assessment of emotional competencies; TEOSQ, task ego orientation sport questionnaire; TOPS, test of performance strategies; TOQS, thought occurrence questionnaire sport; WC, waiting-list control group

## Footnotes

1 Also referred to as behavioral assessment or real life measurement.

2 A curling game has either eight or ten ends (i.e., game sections), and it happens that team forfeits during the game.

3 To win a volleyball game, three winning sets are needed (two in beach volleyball), so the minimum amount of sets in one game is three. In beach volleyball we would analyze the first two sets as this is the minimum amount of sets in one game.

4 A tennis match contains a minimum of two sets; to win a set, a player needs to win six games and players take a first short break after three games.

5 A game in hockey consists of three periods.

6 Other issues involve handling high training loads, improving training quality, fulfilling psychosocial development, or cooperating with various people (see Birrer and Morgan [5] for an overview of the demands athletes need to deal with).
